# Inhibition of the JAK2/STAT3 pathway and cell cycle re‐entry contribute to the protective effect of remote ischemic pre‐conditioning of rat hindlimbs on cerebral ischemia/reperfusion injury

**DOI:** 10.1111/cns.14023

**Published:** 2022-11-23

**Authors:** Yongmei Zhao, Mao Ding, Feng Yan, Jie Yin, Wenjuan Shi, Nan Yang, Haiping Zhao, Yalan Fang, Yuyou Huang, Yangmin Zheng, Xueqi Yang, Wei Li, Xunming Ji, Yumin Luo

**Affiliations:** ^1^ Institute of Cerebrovascular Disease Research Xuanwu Hospital of Capital Medical University Beijing China; ^2^ Beijing Geriatric Medical Research Center and Beijing Key Laboratory of Translational Medicine for Cerebrovascular Diseases Beijing China; ^3^ Beijing Institute for Brain Disorders Capital Medical University Beijing China

**Keywords:** cerebral ischemia/reperfusion, cyclin D1, cyclin‐dependent kinases 6, Janus‐activated kinase 2/signal transducer and activator of transcription 3, remote ischemic pre‐conditioning

## Abstract

**Aims:**

Remote ischemic pre‐conditioning (RIPC) protects against ischemia/reperfusion (I/R) injury. However, the mechanisms underlying this protection remain unclear. In the present study, we investigated the role of Janus‐activated kinase 2 (JAK2)/signal transducer and activator of transcription 3 (STAT3) pathway and cell cycle arrest, and their relationship with neuronal apoptosis following RIPC.

**Methods:**

A rat cerebral I/R injury model was induced by middle cerebral artery occlusion (MCAO), and AG490 was used to investigate the mechanisms of RIPC. p‐JAK2‐, p‐STAT3‐, cyclin D1‐, and cyclin‐dependent kinase 6 (CDK6) expression was assessed by Western blotting and immunofluorescence staining.

**Results:**

RIPC reduced the infarct volume, improved neurological function, and increased neuronal survival. Furthermore, p‐JAK2 and p‐STAT3 were detected during the initial phase of reperfusion; the expression levels were significantly increased at 3 and 24 h after reperfusion and were suppressed by RIPC. Additionally, the MCAO‐induced upregulation of the cell cycle regulators cyclin D1 and CDK6 was ameliorated by RIPC. Meanwhile, cyclin D1 and CDK6 were colocalized with p‐STAT3 in the ischemic brain.

**Conclusion:**

RIPC ameliorates the induction of the JAK2/STAT3 pathway and cell cycle regulators cyclin D1 and CDK6 by MCAO, and this net inhibition of cell cycle re‐entry by RIPC is associated with downregulation of STAT3 phosphorylation.

## Graphical abstract

Three days’ RIPC mitigates I/R injury by decreasing the induction of JAK2/STAT3 pathway and cell cycle regulators cyclin D1 and CDK6 by MCAO, and this net inhibition of cell cycle re‐entry by RIPC is associated with downregulation of STAT3 phosphorylation.

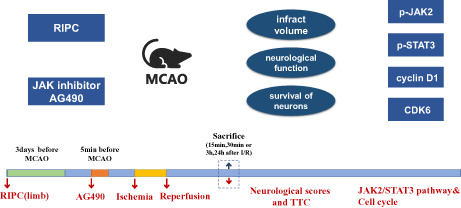

## 1 INTRODUCTION

Stroke is a leading cause of death worldwide and is associated with long‐term disability.^1^ However, the clinical treatment of stroke is limited. Remote ischemic pre‐conditioning (RIPC) is ischemia that is performed in one organ and protects against subsequent prolonged ischemia in another distant organ.^2^ RIPC has greater potential for clinical application than conventional preconditioning because it can be performed in a non‐vital organ. Remote preconditioning of the ipsilateral hindlimb protects against ischemic damage after focal cerebral ischemia in rats.^3^ We previously demonstrated that limb RIPC exerted neuroprotective effects against ischemia using an intraluminal thread middle cerebral artery occlusion (MCAO) and reperfusion model in rats.^4^ However, the endogenous neuroprotective mechanisms of RIPC remains unclear.

The Janus‐activated kinase 2 (JAK2)/signal transducer and activator of transcription 3 (STAT3) pathway is involved in many physiological processes, including those governing cell survival, inflammation, development, proliferation, and differentiation.^5^ Activation of this pathway occurs when a hormone, growth factor, or cytokine binds to a receptor that then activates JAK2 and phosphorylates STAT3. Several groups have reported that the JAK2/STAT3 pathway is activated in in vitro and in vivo experimental models of stroke.^6^, ^7^, ^8^ However, the precise role of JAK2/STAT3 activation after stroke remains unclear. The genes involved in apoptosis are regulated by STAT signaling.^9^ Different apoptosis‐ or antiapoptosis‐related genes can also be transcriptionally modulated via JAK/STAT upon upstream receptor activation.^10^, ^11^ In a hypoxic–ischemic brain injury model, granulocyte colony‐stimulating factor treatment was associated with increased STAT3 and Bcl‐2 expression, which induced antiapoptotic effects.^12^ In contrast, some studies have suggested that the phosphorylation of STAT3 contributes to neuronal apoptotic death after cerebral ischemia.^13^, ^14^ Currently, however, it is unknown whether the JAK2/STAT3 signal pathway is involved in RIPC‐produced neuroprotection against ischemia/reperfusion (I/R) injury in rats.

The cell cycle is a highly coordinated process regulated by the appropriate and timely activation of cyclin‐dependent kinases (CDKs). Terminally differentiated neurons irreversibly escape from the cell cycle. However, neurons re‐enter the cell cycle after I/R.^15^, ^16^ Cyclin D1 plays a critical role in cell cycle progression in proliferating cells by activating CDK2, CDK4, or CDK6.^17^ It was demonstrated that cyclin D1 and CDK6 levels increased following cerebral ischemia.^18^, ^19^ Administration of the CDK inhibitor flavopiridol blocks neuronal death and reduces the infarct volume after reperfusion.^20^ Thus, aberrant cell cycle activation is thought to cause apoptosis in post‐mitotic neurons after cerebral ischemia.^19^ Cyclin D1 and CDK6 downregulation may protect neurons from entering and/or progressing through the cell cycle thus preventing neuron damage after ischemia.

Cyclin D1 is an important target gene of STAT3. High cyclin D1 expression was observed in laryngeal carcinomas, and the positive correlation between phosphorylated STAT3 (p‐STAT3) protein and cyclin D1 mRNA suggests that STAT3 promotes cyclin D1 transcription in laryngeal carcinogenesis.^21^ The expression of cyclin D1 is regulated by STAT3 by binding to its promoter, and a constitutively active STAT3 construct can upregulate cyclin D1 expression at the transcriptional level in rodent fibroblast cell lines.^22^, ^23^ JAK2/STAT3 pathway inhibition suppressed the expression of cyclin D1, cyclin E, and CDK4 in YD‐38 gingival cancer cells.^24^ However, it is unclear whether RIPC inhibits the JAK2/STAT3 pathway and cyclin D1 and CDK6 following I/R injury.

In this study, we investigated whether RIPC could prevent the expression and activation of the JAK2/STAT3 pathway and cyclin D1/CDK6 and aimed to determine the relationship between JAK2/STAT3 and cyclin D1/CDK6 in neurons, as well as their relationship with neuronal apoptosis during cerebral I/R. We hypothesized that RIPC may exert its beneficial effects by intervening in the activation of the JAK2/STAT3 pathway and cell cycle re‐entry in ischemic neurons.

## 2 MATERIALS AND METHODS

### 2.1 Rat model of focal cerebral I/R

Male Sprague–Dawley rats weighing 280–300 g were purchased from Vital River Laboratory Animal Technology Co. Ltd. (Beijing). The animal protocols for these studies were approved by the Institutional Animal Care and Use Committee of Xuanwu Hospital of Capital Medical University. The rats were anesthetized with 3.5% enflurane in N_2_O:O_2_ (70%:30%). Physiological monitoring during the procedure comprised measurement of the rectal temperature, mean arterial blood pressure, and heart rate. The rats were subjected to MCAO followed by reperfusion using a suture occlusion model, as previously described.^25^ The animals underwent right MCAO for 90 min and were then reperfused for 15 min, 30 min, 3 h, and 24 h after withdrawal of the filament. Successful MCAO was assessed by circling to the non‐ischemic side (left) at the end of ischemia and further confirmed by 2,3,5‐triphenyltetrazolium chloride (TTC) staining at the end of reperfusion.

### 2.2 RIPC, drug administration, and experimental groups

RIPC was performed by occluding the bilateral femoral arteries with aneurysm clips (FE 681 K, Aesculap Inc.) for 10 min, followed by 10 min of reperfusion. This was considered as one cycle. For preconditioning, this cycle was repeated thrice. Three cycles of RIPC were administered once per day for 3 days before the animals underwent MCAO and reperfusion. The JAK2 inhibitor AG490 (40 mg/kg dissolved in 3% DMSO) was injected intraperitoneally 5 min before reperfusion. All other groups received 3% DMSO as a control.

Rats were randomly assigned to five groups: (1) vehicle‐treated sham‐operated (Sham), (2) RIPC‐treated sham (Sham+R), (3) vehicle‐treated MCAO (Vehicle), (4) RIPC‐treated MCAO (RIPC), and (5) AG490 + RIPC‐treated MCAO (AG490 + R). Each group was further divided into four subgroups according to the reperfusion time after 90 min MCAO, that is, 15 min, 30 min, 3 h, and 24 h (*n* = 8 in each subgroup). Animals exhibiting convulsions or sustained consciousness disturbances were excluded from subsequent experiments. Five rats with unsuccessful MCAO were excluded from the study. No rats died from stroke complications.

### 2.3 Behavioral tests

Neurological deficits were assessed at 3 and 24 h after reperfusion in a double‐blind fashion using the Ludmila Belayev and Zea Longa five‐point scoring systems. For the Ludmila Belayev score, neurological function was graded on a scale of 0–12, with 0 representing normal function and 12 representing maximum neurological deficits.^26^ For the Zea Longa five‐point scoring system, neurological deficits were scored using a modified scoring system developed by Longa et al.^27^

### 2.4 Tissue collection and infarct volume determination

At the end of reperfusion, the rats were exposed to 5% isoflurane (in oxygen) and decapitated after ensuring that the animal was completely unresponsive to tail pinch. The brains were harvested and sectioned into four 2‐mm‐thick coronal slices from an 8‐mm‐thick region 5 mm away from the tip of the frontal lobe. The first and third slices were prepared for Western blot analysis. The second and fourth slices were incubated in 1% (w/v) TTC (Sigma‐Aldrich) solution at 37°C for 20 min to measure the infarct volume^25^ by blinded investigators. The remaining brain tissues were stored at −80°C. Coronal brain sections (20 μm) were obtained using a cryostat (CM1900, Leica) for histological staining.

The infarct volume was measured using ImageJ analysis software, as described previously.^25^ To minimize the error introduced by edema, an indirect method for calculating the infarct volume was used.^28^ The infarct volume was presented as a percentage of the volume of the contralateral hemisphere.

### 2.5 Examination of apoptotic cell death

To identify apoptotic cell death in the brain sections, terminal deoxyribonucleotide transferase dUTP nick‐end labeling (TUNEL) staining was performed using an in situ cell death detection kit, according to the manufacturer's instructions (Roche). Briefly, after washing in phosphate‐buffered saline (PBS), sections were incubated with reaction buffer containing terminal deoxynucleotidyl transferase at 37°C for 60 min in the dark. The nuclei were stained with 4′,6‐diamidino‐2‐phenylindole (DAPI), and sections were observed under a fluorescence microscope (80i; Nikon).

### 2.6 Western blotting analysis

P‐JAK2 and p‐STAT3 protein expression was analyzed by Western blotting. The ipsilateral hemisphere was homogenized in lysis buffer containing protease inhibitors, as previously described.^25^ The nuclear proteins were lysed using a nuclear protein extraction kit (Beyotime), according to the manufacturer's instructions. Protein content was determined using a bicinchoninic acid protein assay; 40 μg of protein was electrophoresed in 8% SDS‐PAGE and transferred to a polyvinylidene fluoride membrane. The membranes were probed with p‐JAK2, JAK2, p‐STAT3 (Tyr705), and STAT3 antibodies. All the antibodies were from Cell Signaling Technology. Quantitative results were obtained by measuring the optical density of each band and expressed as the ratio of each targeted protein to β‐actin or JAK2 and STAT3 expression.

### 2.7 Immunofluorescence analysis

Sections were treated with either p‐JAK2 antibody, p‐STAT3 antibody, cyclin D1 antibody (Cell Signaling Technology), CDK6 antibody (Cell Signaling Technology) with neuron‐specific nuclear protein (NeuN) antibody (Chemicon), glial fibrillary acidic protein (GFAP) antibody (Santa), or ionized calcium‐binding adapter molecule‐1 (Iba‐1) antibody (Cell Signaling Technology); either cyclin D1 antibody or CDK6 antibody with p‐STAT3 antibody at 4°C overnight. The sections were then incubated with a secondary antibody (Invitrogen) for 1 h at 22 ± 2°C. The nuclei were stained with DAPI.

For double staining of NeuN, GFAP, Iba‐1, cyclin D1, or CDK6 and TUNEL, slices were incubated with the primary antibodies against NeuN, GFAP, Iba‐1, cyclin D1, or CDK6, respectively, at 4°C overnight, with PBS used as a negative control. Following three washes in PBS, the sections were incubated with fluorescent‐labeled secondary antibodies. Sections were then stained using the TUNEL assay kit according to the manufacturer's instructions and counterstained with DAPI.

### 2.8 Statistical analysis

The results are reported as the mean ± standard deviation. Statistical analysis was performed using SPSS version 20.0 (SPSS). The Shapiro–Wilk normality test was used to analyze the normality of data, and the data were found to be normally distributed. The difference between means was evaluated using one‐way analysis of variance (ANOVA) and post hoc least significant difference/Tamhane T2 tests for multiple comparisons, with *p* < 0.05 indicating statistically significance.

## 3 RESULTS

### 3.1 RIPC reduced the infarct volume and improved neurological dysfunction in ischemic rats

**FIGURE 1:**
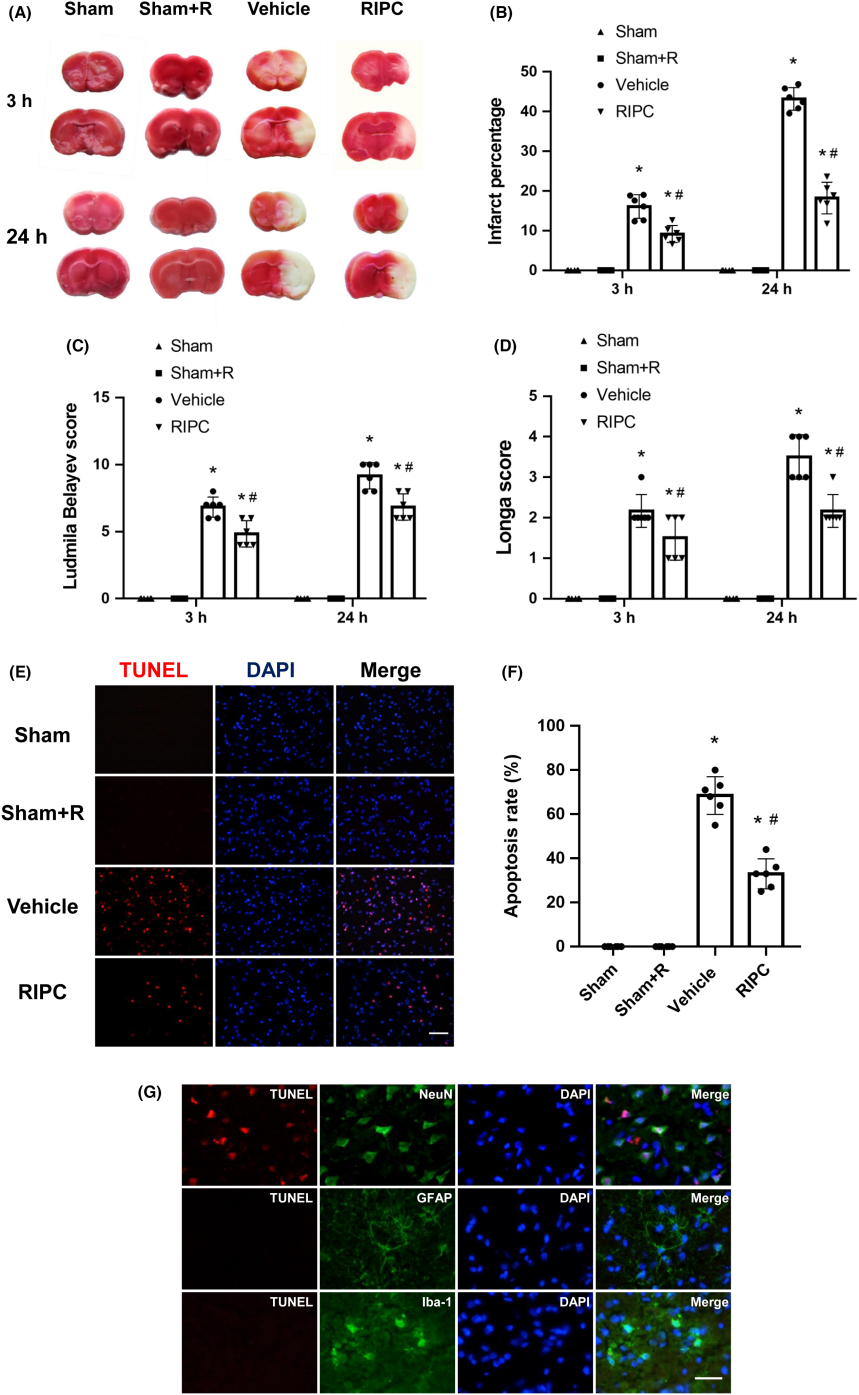
Remote ischemic pre‐conditioning (RIPC) treatment reduces ischemia/reperfusion injury and neuronal apoptosis in middle cerebral artery occlusion (MCAO) rats. (A) Cerebral infarction area was measured using 2,3,5‐triphenyltetrazolium chloride (TTC) staining. (B) RIPC treatment reduces brain tissue loss in MCAO rats. RIPC treatment improves neurobehavioral outcome in MCAO rats as evidenced by the Ludmila Belayev score (C) and Longa score (D). (E) Representative staining for terminal deoxyribonucleotide transferase dUTP nick end labeling (TUNEL) (red) of brain slices from different groups at 24 h after reperfusion. Bar = 50 μm. (F) Quantification of TUNEL‐positive cells. Data are presented as the means ± standard deviations. Significant differences were evaluated using one‐way analysis of variance followed by the Tamhane T2 test. *p < 0.05 vs the sham group; ^#^ *p* < 0.05 vs the vehicle‐treated MCAO group. (G) Colocalization of TUNEL (red) and neuron‐specific nuclear protein (NeuN) (green), glial fibrillary acidic protein (GFAP) (green), or ionized calcium‐binding adapter molecule‐1 (Iba‐1) (green) in the ischemic penumbra of MCAO rats at 24 h after reperfusion. Bar = 20 μm. Nuclei were counterstained with 4′,6‐diamidino‐2‐phenylindole (DAPI) (blue).

To confirm the neuroprotective effect of RIPC on cerebral ischemic injury, we compared the severity of brain damage with or without RIPC treatment after 3 and 24 h of reperfusion following ischemia. The infarct volume and neurological scores were calculated to measure brain damage (Figure 1A–D). Quantitative analysis revealed that cerebral I/R significantly increased the infarct volume in ischemic hemispheres, whereas sham control rats did not exhibit any lesions in either hemisphere. RIPC treatment markedly reduced the infarct volume in the ischemic hemisphere at 3 and 24 h after reperfusion (*p* < 0.05). This marked reduction in infarct volume could not be attributed to hypothermia or other alterations in physiological parameters, such as rectal temperature, mean arterial blood pressure, and heart rate, which did not differ between the experimental groups. Neurological function was assessed using the Ludmila Belayev and Longa scores at 3 and 24 h following ischemia. RIPC treatment significantly decreased the scores after ischemic stroke compared with those in the vehicle‐treated group (*p* < 0.05), confirming the protective effect of RIPC on ischemic brain damage in rats. To delineate the effect of RIPC on a non‐stroke background, the Sham+R group was used. There was no significant difference in infarct volume and neurological function between the Sham and Sham +R groups.

### 3.2 RIPC reduced neuronal apoptotic death in rats following cerebral ischemia

To study the effect of RIPC on apoptosis following transient focal cerebral ischemia, TUNEL staining was used to stain apoptotic cells in rat brain sections. As illustrated in Figure 1E,F, TUNEL staining was not observed in the brains of Sham and Sham+R group rats, while the number of TUNEL‐positive cells was increased in the brains of MCAO group rats at 24 h after reperfusion. Moreover, TUNEL‐positive cells were largely colocalized with NeuN, a marker of neuronal maturation but were not colocalized with astrocytes or microglia (Figure 1G), suggesting that cerebral ischemia mainly induced neuronal apoptotic death after 24 h of reperfusion. Interestingly, fewer TUNEL‐positive cells were observed in RIPC‐treated MCAO rats than in MCAO rats after 24 h of reperfusion (*p* < 0.05, Figure 1E,F). This result indicated that 3 days of RIPC before MCAO ameliorated apoptotic programmed neuronal death following transient focal cerebral ischemia.

### 3.3 RIPC reduced JAK2 and STAT3 phosphorylation in ischemic brains

**FIGURE 2:**
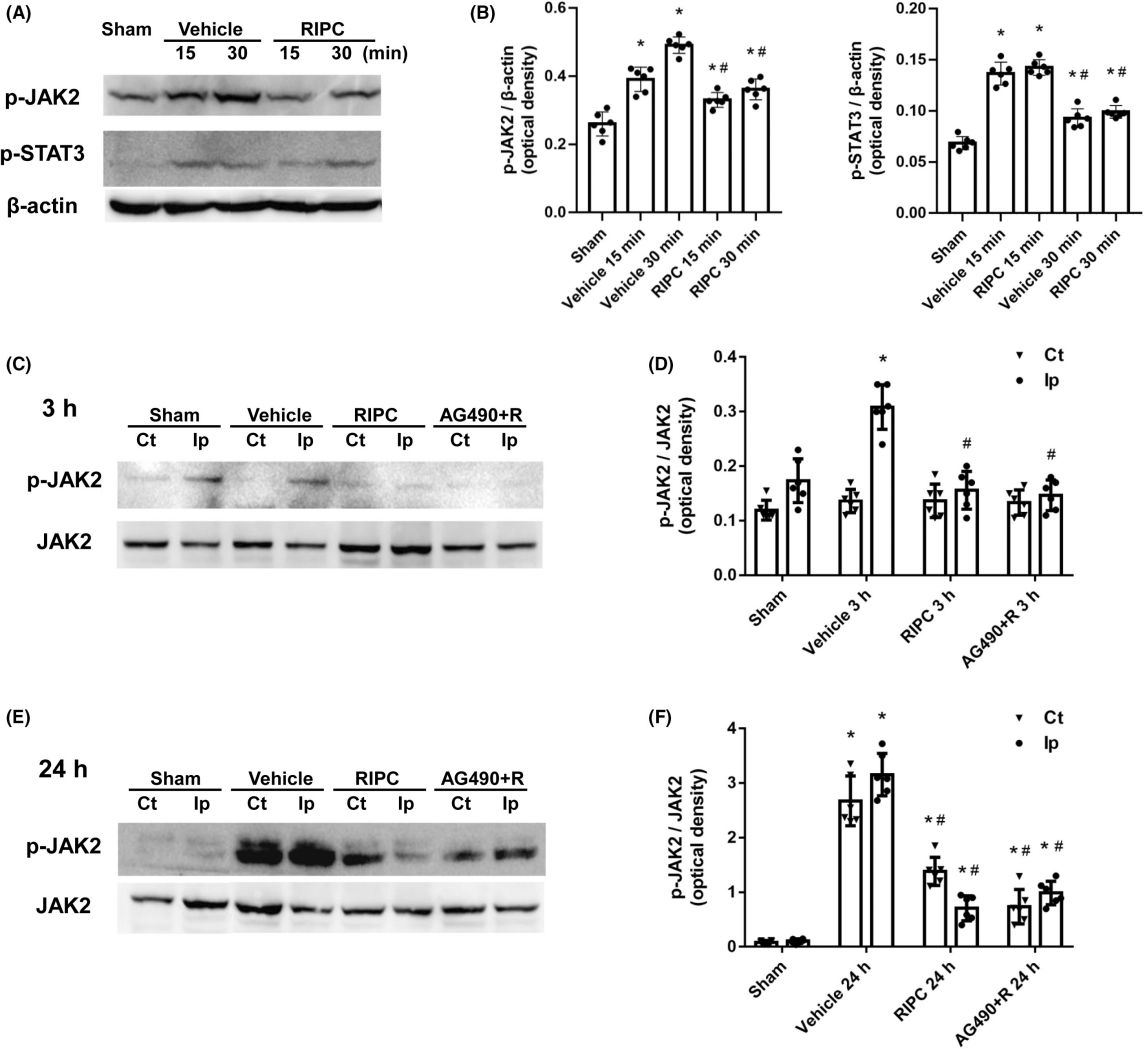
Remote ischemic pre‐conditioning (RIPC) treatment reduces the phosphorylation of Janus‐activated kinase 2 (JAK2) and signal transducer and activator of transcription 3 (STAT3) in the ischemic hemisphere of middle cerebral artery occlusion (MCAO) rats. (A, B) Brain tissue was isolated 15 and 30 min after reperfusion and examined for the expression of phosphorylated JAK2 (p‐JAK2) and phosphorylated STAT3 (p‐STAT3) by Western blotting, with β‐actin used as a loading control. (C, D) Brain tissue was isolated 3 h and (E, F) 24 h after reperfusion and examined for the expression of p‐JAK2 by Western blotting, with total JAK2 used as a loading control. Data are expressed as means ± standard deviation. Significant differences were evaluated using one‐way analysis of variance followed by the post hoc least significant difference test (A, B) and the Tamhane T2 test (C–F). *p < 0.05 vs the sham group, ^#^ *p* < 0.05 vs the vehicle‐treated MCAO group. Ct, contralateral hemisphere; Ip, ipsilateral hemisphere.

**FIGURE 3:**
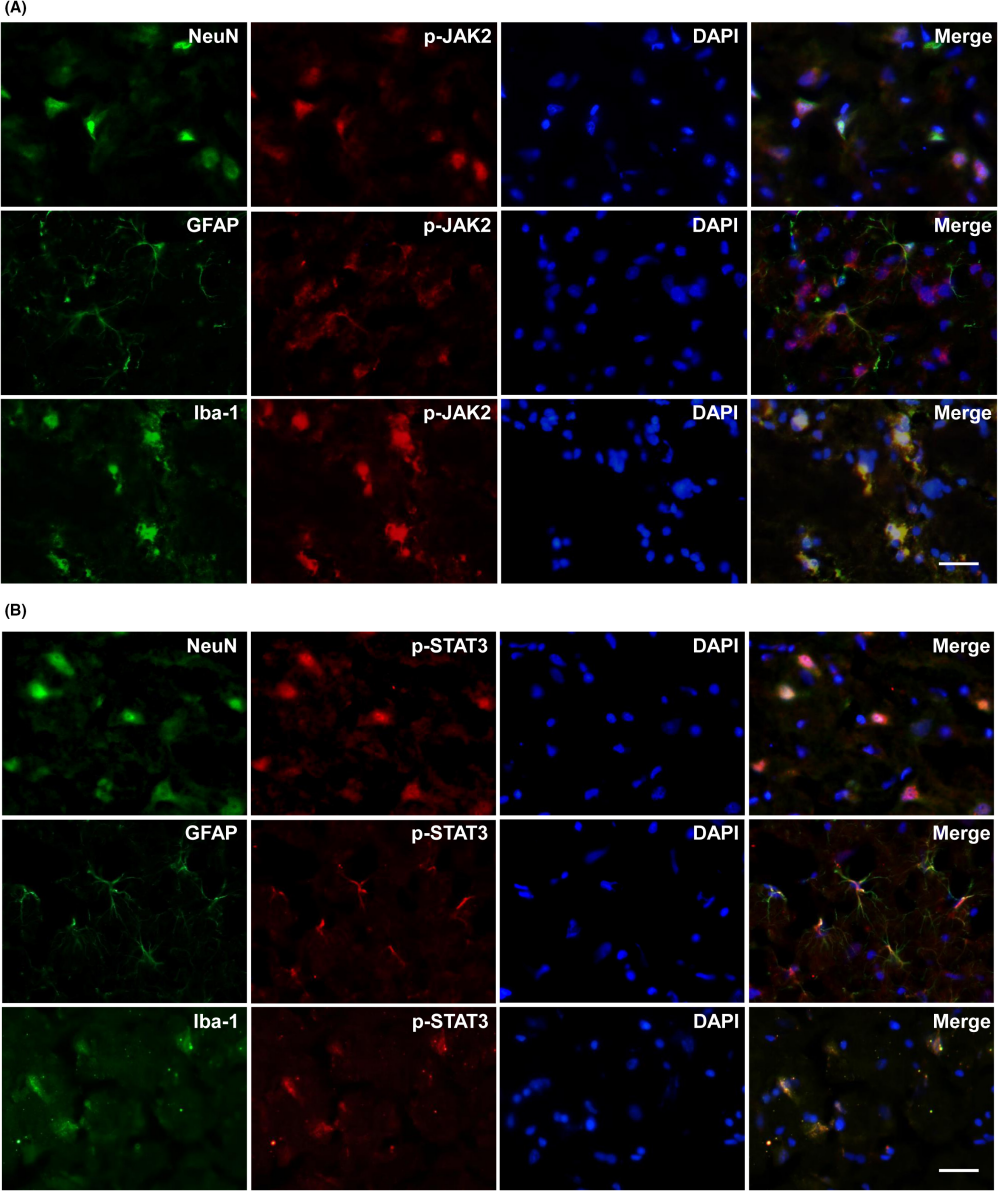
Phosphorylated Janus‐activated kinase 2 (p‐JAK2) and phosphorylated signal transducer and activator of transcription 3 (p‐STAT3) immunofluorescence‐stained cells appear in neurons, astrocytes, and microglia in the ischemic penumbra of middle cerebral artery occlusion (MCAO) rats 24 h after reperfusion. (A) Colocalization of p‐JAK2 (red) or (B) p‐STAT3 (red) with neuron‐specific nuclear protein (NeuN) (green), glial fibrillary acidic protein (GFAP) (green), or ionized calcium‐binding adapter molecule‐1 (Iba‐1) (green) in the ischemic penumbra of MCAO rats. Bars = 20 μm. Nuclei were counterstained with 4′,6‐diamidino‐2‐phenylindole (DAPI) (blue).

To explore the mechanism of RIPC‐induced neuroprotection, we investigated whether RIPC regulates JAK2 and STAT3 activation in MCAO rats. The activation of JAK2 and STAT3 was measured by Western blotting and immunofluorescence. As shown in Figure 2, p‐JAK2 and p‐STAT3 in MCAO rats were detected at 15 and 30 min after reperfusion (Figure 2A,B), indicating that the JAK2/STAT3 pathway might be activated in the early stage of reperfusion after MCAO. At 3 and 24 h after reperfusion, faint immunoreactivity for p‐JAK2 was detected in the ipsilateral hemisphere after sham surgery but was significantly increased in the ischemic hemisphere of rats subjected to MCAO (*p* < 0.05, Figure 2C–F). The level of p‐JAK2 in the ischemic hemisphere was significantly reduced in RIPC rats compared MCAO rats (*p* < 0.05, Figure 2A–F). Double immunofluorescent staining using NeuN, GFAP, and Iba‐1 markers confirmed that p‐JAK2 and p‐STAT3 immunofluorescence was observed in neurons, astrocytes, and microglia after cerebral I/R (Figure 3A,B). However, TUNEL staining only occurred in neurons after 24 h of reperfusion (Figure 1G). Thus, the antiapoptotic effect of RIPC in neurons could be achieved by JAK2/STAT3 pathway inhibition in MCAO rats. However, pretreatment with 30 μM of AG490, a JAK2‐specific inhibitor, did not further significantly reduce the ischemia‐induced phosphorylation of JAK2 at 3 and 24 h after reperfusion compared with RIPC treatment (Figure 2C–F). These findings further confirmed that the regulatory effect of RIPC on the JAK2/STAT3 pathway is similar to that of AG490.

### 3.4 RIPC inhibits the nuclear translocation of p‐STAT3 following cerebral I/R

**FIGURE 4:**
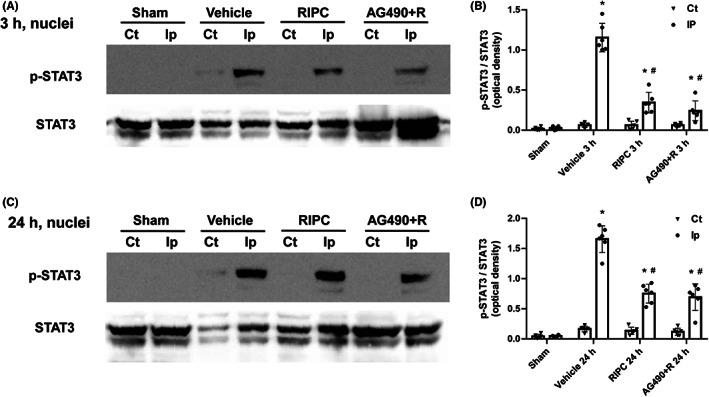
Remote ischemic pre‐conditioning (RIPC) treatment inhibits the nuclear translocation of phosphorylated signal transducer and activator of transcription 3 (p‐STAT3) in the ischemic hemisphere of middle cerebral artery occlusion (MCAO) rats. (A, B) Nuclear extracts were prepared from the cerebral hemisphere of MCAO rats at 3 h and (C, D) 24 h after reperfusion and examined for the expression of p‐STAT3 by Western blotting, with total STAT3 used as a loading control. Data are expressed as means ± standard deviation. Significant differences were evaluated using one‐way analysis of variance followed by the Tamhane T2 test. *p < 0.05 vs the sham group, ^#^ *p* < 0.05 vs the vehicle‐treated MCAO group. Ct, contralateral hemisphere; Ip, ipsilateral hemisphere.

Upon tyrosine phosphorylation triggered by diverse stimuli, STAT3 dimerizes and translocates to the nucleus to induce gene transcription.^29^ Its role as a DNA‐binding transcription factor depends on its ability to enter the nucleus. Therefore, under the present experimental conditions, we extracted the nuclear fraction of MCAO rats 3 and 24 h after reperfusion and determined whether the neuroprotection afforded by RIPC was associated with changes in nuclear p‐STAT3 levels in the brains of rats subjected to I/R. Ischemic insult significantly increased the levels of STAT3 phosphorylation in the nuclear fractions of the ipsilateral ischemic brain hemisphere at 3 and 24 h after reperfusion (*p* < 0.05, Figure 4A–D). However, in comparison with vehicle‐treated animals, RIPC reduced STAT3 phosphorylation in the nuclear fraction 3 and 24 h after ischemia (*p* < 0.05). In addition, pre‐treatment with AG490 did not further reduce the ischemia‐induced phosphorylation of STAT3 in nuclear fractions at 3 and 24 h after reperfusion. Immunofluorescence staining showed that there were increased p‐STAT3‐positive cells in the brain of MCAO group rats, which decreased significantly, however, in the RIPC group rats at 24 h after reperfusion. In addition, there was no significant difference in p‐JAK2 and p‐STAT3 expression between the Sham and Sham + R groups (Figure S1). These results indicate that I/R induced the nuclear translocation of p‐STAT3 in the ischemic hemisphere, which was attenuated by RIPC treatment.

### 3.5 Cell cycle regulators cyclin D1 and CDK6 are associated with the phosphorylation of STAT3 in ischemic neurons

**FIGURE 5:**
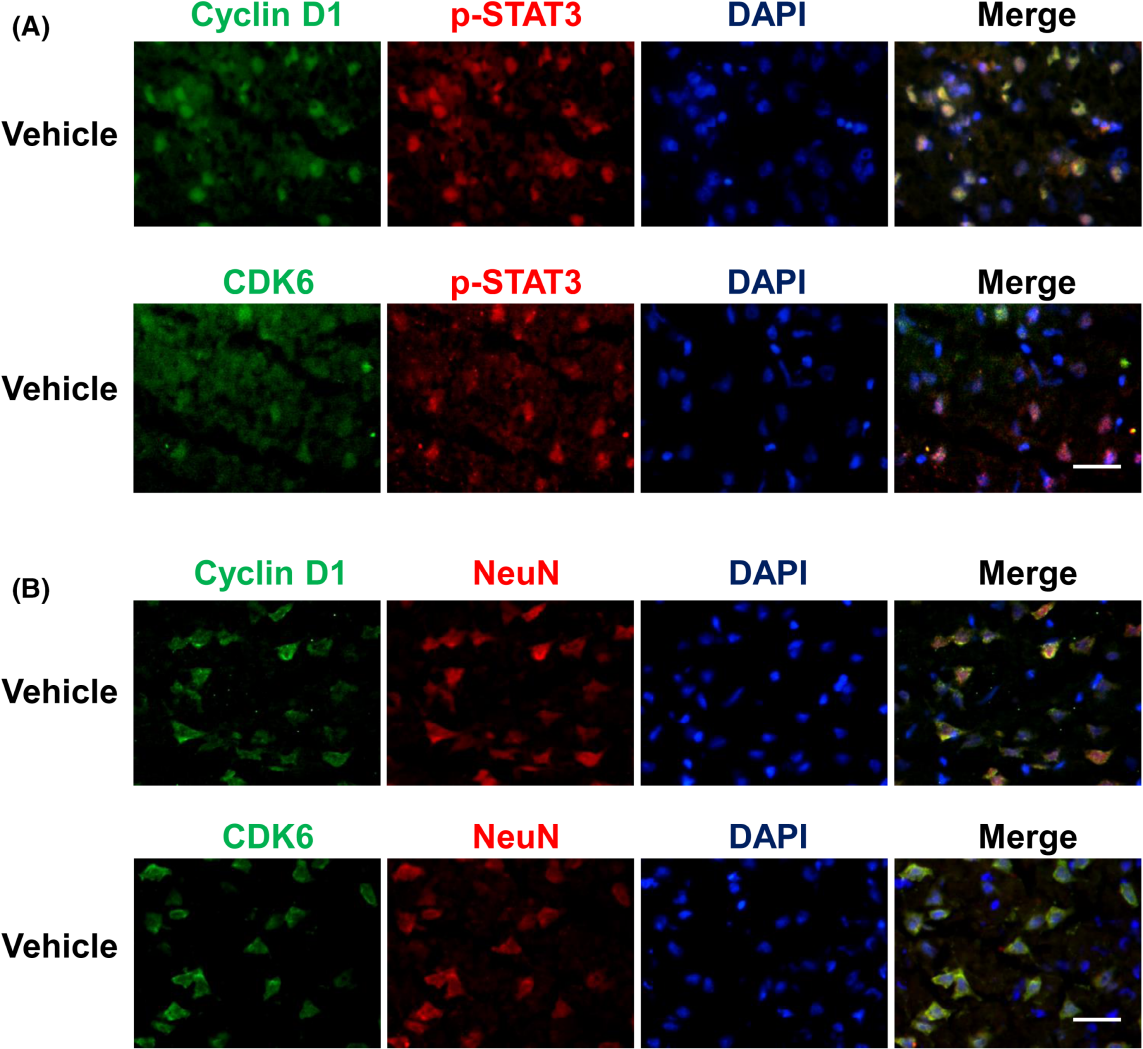
Cyclin D1‐ and cyclin‐dependent kinases 6 (CDK6) in neurons are associated with the phosphorylation of signal transducer and activator of transcription 3 (STAT3) in the ischemic penumbra of middle cerebral artery occlusion (MCAO) rats 24 h after reperfusion. (A) Colocalization of cyclin D1 (green) or CDK6 (green) with phosphorylated STAT3 (p‐STAT3) (red) in the ischemic penumbra of MCAO rats. (B) Colocalization of cyclin D1 (green) or CDK6 (green) with neuron‐specific nuclear protein (NeuN) (red) in the ischemic penumbra of MCAO rats. Bars = 20 μm. Nuclei were counterstained with 4′,6‐diamidino‐2‐phenylindole (DAPI) (blue).

It has been reported that STAT3 transfers to the nucleus to control the transcription of the target downstream gene cyclin D1.^30^ To determine whether the JAK2/STAT3 pathway is involved in cell cycle arrest in neurons, we utilized double immunofluorescence staining to detect cyclin D1/CDK6 positivity and the expression of p‐STAT3. Figure 5A shows that cyclin D1/CDK6‐positive cells were colocalized with p‐STAT3 in the ischemic penumbra of MCAO rats 24 h after reperfusion, indicating that the upregulation of cyclin D1/CDK6 is closely associated with STAT3 phosphorylation. In addition, double immunofluorescence staining showed that cyclin D1/CDK6‐positive cells colocalized with NeuN (Figure 5B), suggesting cyclin D1/CDK6 upregulation in neurons after MCAO. These results verified that cell cycle re‐entry occurred within neurons after MCAO, and this re‐entry was associated with the phosphorylation of STAT3 in neurons during ischemia injury.

### 3.6 The upregulations of cyclin D1 and CDK6 is associated with neuronal apoptosis in the ischemic brain and is suppressed by RIPC

**FIGURE 6:**
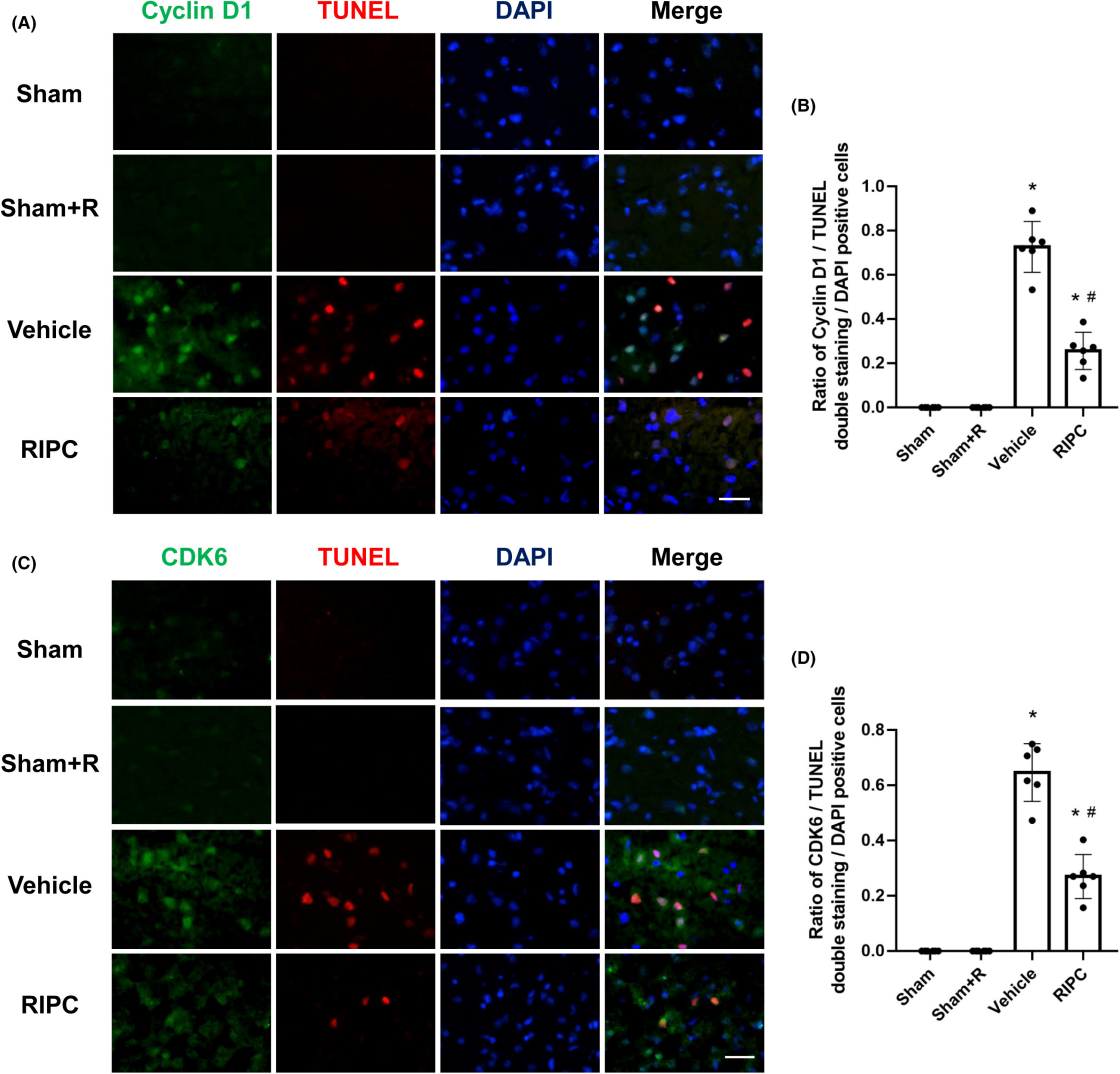
Remote ischemic pre‐conditioning (RIPC) suppresses cyclin D1‐/cyclin‐dependent kinases 6 (CDK6)‐associated cell apoptosis in the ischemic penumbra of middle cerebral artery occlusion (MCAO) rats 24 h after reperfusion. (A) Representative double staining for cyclin D1 (green) and terminal deoxyribonucleotide transferase dUTP nick end labeling (TUNEL) (red) in the ischemic penumbra of MCAO rats. (B) Quantification of cyclin D1/TUNEL positive cells. (C) Representative double staining for CDK6 (green) and TUNEL (red) in the ischemic penumbra of MCAO rats. Bars = 20 μm. Nuclei were counterstained with 4′,6‐diamidino‐2‐phenylindole (DAPI) (blue). (D) Quantification of CDK6/TUNEL positive cells. Data are presented as the means ± standard deviation. Significant differences were evaluated using one‐way analysis of variance followed by the Tamhane T2 test. *p < 0.05 vs the sham group; ^#^ *p* < 0.05 vs the vehicle‐treated MCAO group.

Neuronal cell cycle re‐entry is proapoptotic. Cell cycle progression of G1/S and G2/M transition is regulated by the synthesis and activity of cyclin and CDK complexes in the concerning phase.^16^ Here, we investigated whether MCAO could induce the upregulation of two positive cell cycle regulators, cyclin D1 and CDK6, which drive G1/S transition, and whether this upregulation could be inhibited by RIPC treatment. Immunofluorescence staining identified numerous cyclin D1‐positive and CDK6‐positive cells in the ischemic penumbra of the MCAO group rats. Double immunofluorescence staining revealed that cyclin D1/CDK6 was co‐localized with TUNEL‐positive cells in the penumbral tissue of vehicle‐treated MCAO rats after 90 min of ischemia and 24 h of reperfusion (Figure 6A–D), indicating that ischemia‐induced neuronal apoptosis is closely associated with the upregulation of cyclin D1 and CDK6 expression. RIPC treatment reduced the cyclin D1/TUNEL and CDK6/TUNEL double‐positive fractions in MCAO rats (*p* < 0.05), while there was no significant difference between the Sham and Sham+R groups (Figure 6A–D). These results indicate that I/R‐induced neuronal apoptotic death is associated with the high expression of the cell cycle regulators cyclin D1 and CDK6 and that RIPC exerts protective effects by alleviating cell cycle re‐entry.

## 4 DISCUSSION

The protective function of RIPC in cerebral ischemia is widely recognized.^31^, ^32^, ^33^ In this study, we used an MCAO model to simulate the process of cerebral I/R to verify the effects of RIPC on cerebral I/R and explore its mechanism of action. We found that RIPC reduces the brain infarct damage and neurological deficits produced by focal cerebral I/R injury in rats after 3 and 24 h of reperfusion. Accordingly, the number of apoptotic neurons decreased after RIPC. Further, p‐JAK2 and p‐STAT3 levels were significantly elevated in the ipsilateral brain hemisphere 15 min to 24 h after reperfusion. RIPC treatment inhibits the activation of JAK2/STAT3 signaling molecules in the ischemic brain, indicating that the neuroprotective effect of RIPC occurs via the modulation of JAK2 and STAT3 phosphorylation. In addition, we identified the occurrence of cell cycle re‐entry in the ischemic penumbra of MCAO rats and provided initial evidence that RIPC significantly attenuates MCAO‐induced cell cycle re‐entry. Importantly, the cell cycle regulators cyclin D1 and CDK6 were found to be closely associated with STAT3 phosphorylation in neurons.

Studies on ischemia reperfusion injury of the heart,^34^ liver,^35^ kidney,^36^ and intestines^29^ have implicated JAK2/STAT3 as a key membrane‐to‐nucleus signaling pathway that responds to diverse stresses. Focal ischemia‐induced STAT3 phosphorylation was previously reported to be localized in various cell types, including microglia/macrophages, astrocytes, and neurons.^6^, ^7^, ^8^ In the present study, we observed the cellular localization of p‐JAK2 and p‐STAT3 in all of these cell types (Figure 3), confirming the results of previous studies and indicating that I/R injury activates the JAK2/STAT3 pathway in the central nervous system. P‐STAT3 immunoreactivity was first detected 3.5 h after reperfusion in each cortical and striatal region.^37^ The present study showed that p‐JAK2 and p‐STAT3 levels were slightly increased at 15 and 30 min after reperfusion; to the best of our knowledge, this is the first report of p‐JAK2 and p‐STAT3 detection during the initial phase of reperfusion. In our study, the expression of p‐JAK2 and p‐STAT3 increased continuously at 3 and 24 h after reperfusion. STAT3 phosphorylation is important for its function, as the role of STAT3 as a DNA‐binding transcription factor depends on its ability to enter the nucleus.^8^ We also explored the nuclear p‐STAT3 levels in the brain hemisphere. After 3 h of reperfusion, p‐STAT3 was detected in the nuclei and increased in the nuclear 24 h after reperfusion, indicating that p‐STAT3 translocation occurred soon after cerebral ischemia as an early step of activated signal transduction.

Although JAK2/STAT3 pathway activation leads to the increased expression of genes associated with cell proliferation, differentiation, and survival after cerebral ischemia, the function of activated JAK2/STAT3 is controversial; some studies have reported an association with survival,^13^, ^30^, ^38^, ^39^ while others have indicated a relationship with cell death.^13^ In the present study, exposure to RIPC significantly reduced the number of TUNEL‐positive cells in the ischemic brain (Figure 1G), whereas p‐JAK2 and p‐STAT3 were inhibited by RIPC, indicating that the JAK2/STAT3 pathway is involved in the process of ischemia‐induced apoptotic cell death. Interestingly, TUNEL‐positive cells were only detected in neurons following cerebral I/R injury, while neither GFAP‐positive astrocytes nor Iba‐1‐positive microglia showed any TUNEL immunoreactivity, indicating that neurons are the most sensitive cell type in the ischemic brain and RIPC reduces ischemia‐induced neuronal apoptosis. We also found that the JAK2‐specific inhibitor AG490 did not further decrease the ischemia‐induced phosphorylation of JAK2 and STAT3 significantly at 3 and 24 h after reperfusion. These data suggest that RIPC treatment exerts the same neuroprotective effect as the JAK2‐specific inhibitor, AG490, and may share the same neuroprotective mechanism by blocking the activation of the JAK2/STAT3 pathway during ischemic injury.

Ischemia activates JAK2 phosphorylation, which is followed by cytoplasmic STAT3 activation. STAT3 transfers to the nucleus to control transcription of the target downstream gene cyclin D1, which is associated with cell cycle regulation.^40^ STAT3 plays an essential role in the regulation of critical cell cycle components.^41^ Further, recent data indicated that JAK2 inhibition could reduce cyclin D1 expression.^42^ Thus, the JAK2/STAT3 pathway is likely to be involved in cell cycle arrest. In the present study, double immunofluorescence staining showed that cyclin D1 and CDK6 were colocalized with p‐STAT3 in the ischemic penumbra, indicating that these cell cycle regulators are associated with STAT3 phosphorylation during ischemia.

In terminally differentiated neurons, the cell cycle is quiescent and does not express many cell‐cycle genes. Evidence for the involvement of cell cycle re‐entry in cerebral ischemia injury indicates that neuronal death after ischemia is often associated with the upregulation and activation of CDKs and cyclins.^43^ We have previously demonstrated that ischemic postconditioning partially reverses cell cycle reactivity following I/R injury.^44^ Here, we investigated whether MCAO could induce the upregulation of cyclin D1 and CDK6, which drive G1/S transition, and whether this upregulation could be inhibited by RIPC treatment. We found that ischemia‐activated cell cycle progression is involved in the process of ischemia‐induced cell apoptosis, as evidenced by the colocalization of enhanced expression of cyclin D1/CDK6 and TUNEL by immunofluorescence staining and that this increased expression was hindered by RIPC treatment. Furthermore, double immunofluorescence staining showed that the majority of cyclin D1‐ and CDK6‐stained cells were NeuN‐positive in the ischemic penumbra, indicating that cell cycle progression occurred in terminally differentiated neurons during cerebral I/R injury. Thus, RIPC may exert neuroprotective effects by attenuating cyclin D1 and CDK6 expression in ischemic neurons, which is associated with reduced STAT3 phosphorylation.

In conclusion, RIPC treatment inhibits the activation of JAK2/STAT3 signaling molecules in the ischemic brain and attenuates the increases in cyclin D1 and CDK6, which would otherwise guide injured neurons into cell cycle re‐entry. JAK2/STAT3 pathway inhibition may be a potential upstream mechanism by which RIPC treatment reduces cyclin D1 and CDK6 expression. These findings are the first to demonstrate the inhibitory effect of RIPC treatment on the JAK2/STAT3 signaling pathway and cell cycle re‐entry in neurons, which provides novel insights for better understanding the mechanisms responsible for the protective effect of RIPC on cerebral ischemic injury.

## CONFLICT OF INTEREST

The authors declare that they have no competing interests. Dr. Yumin Luo is an Editorial Board member of CNS Neuroscience and Therapeutics and a co‐author of this article. To minimize bias, they were excluded from all editorial decision making related to the acceptance of this article for publication.

## Supporting information


Figure S1.

## Data Availability

The data that support the findings of this study are available from the corresponding author upon reasonable request.
