# Supplementary figures and images for: Inhibition of the JAK2/STAT3 pathway and cell cycle re‐entry contribute to the protective effect of remote ischemic pre‐conditioning of rat hindlimbs on cerebral ischemia/reperfusion injury
Source: CNS Neurosci Ther. 2022 Nov 23;29(3):866–77. doi: 10.1111/cns.14023 (PMC9928551; doi:10.1111/cns.14023)

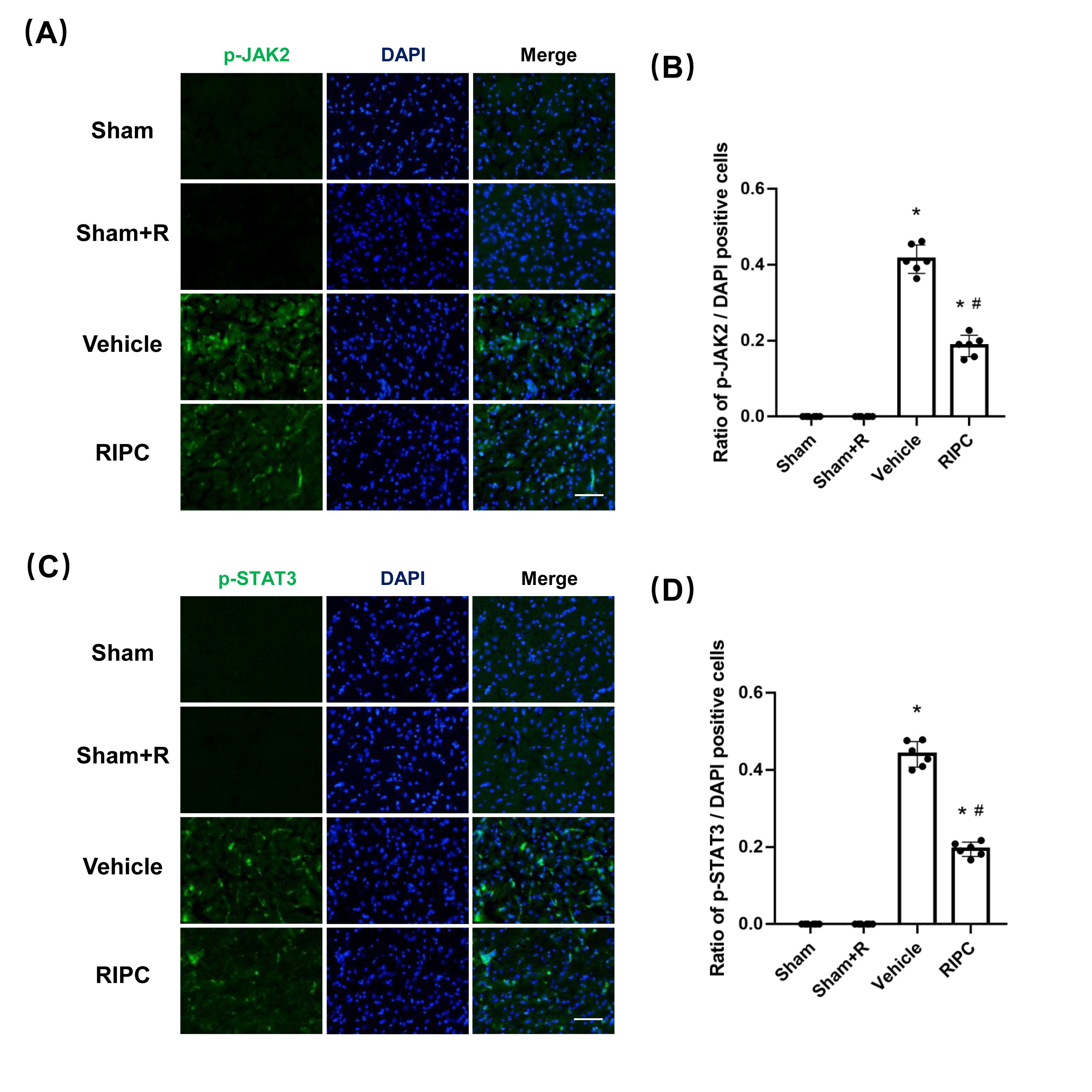

Supplement: Supplementary file 1 — Figure S1. [file CNS-29-866-s001.tif]
